# Associations between clinical and social factors and anticoagulant prescription among patients with atrial fibrillation: A retrospective cohort study from a large healthcare system

**DOI:** 10.1371/journal.pone.0289708

**Published:** 2023-08-10

**Authors:** Rasha Khatib, Nicole Glowacki, Carmine Colavecchia, J. Rebecca Mills, Scott Glosner, Matthew Cato, Peter Brady

**Affiliations:** 1 Advocate Aurora Research Institute, Downers Grove, IL, United States of America; 2 Pfizer Inc, US Medical Affairs, New York, NY, United States of America; 3 Advocate Illinois Masonic Medical Center, Chicago, IL, United States of America; University of Liverpool, UNITED KINGDOM

## Abstract

**Background:**

Patient clinical factors and social determinants of health (SDOH) are associated with an increased risk of stroke for patients with atrial fibrillation (AF); however, the association between these factors and the management of AF is not well characterized, particularly among those factors commonly collected in electronic health records (EHRs). This study used EHR data to evaluate the associations between patient clinical factors and SDOH and prescribing of an oral anticoagulant (OAC) for stroke prevention in AF.

**Methods:**

This analysis included adult patients with newly diagnosed AF who had ≥2 encounters in the Advocate Aurora Health system in Wisconsin between May 2016 and May 2021. Patient-level demographics, comorbidities, medications, and SDOH were retrospectively extracted from EHRs. Area deprivation index (ADI) was linked to patient records as a measure of socioeconomic status.

**Results:**

Of 16,656 patients with AF, 10,898 (65.4%) were prescribed an OAC within the first year of diagnosis. Patients were less likely to be prescribed an OAC (relative risk [95% CI]) if they were widowed (0.98 [0.96–0.99] vs single) or had a history of alcoholism (0.86 [0.79–0.95] vs no history). Most patients (53.3%) received prescriptions from a primary care provider. A linear relationship was found between worsening ADI and increased prescriptions for warfarin vs those for direct-acting OACs.

**Conclusions:**

Although guideline-concordant anticoagulant use remained suboptimal, clinical characteristics were strongly associated for whether a patient with AF would be prescribed an OAC. Disparities in patient care regarding the prescribing of OACs due to SDOH and associated behaviors were small but present, particularly for national ADI.

## Introduction

Atrial fibrillation (AF) is the most commonly experienced heart rhythm disorder and is estimated to affect 2.7 to 6.1 million people in the United States [1,2]. AF is associated with a myriad of complications, including myocardial infarction [3–5] and heart failure [6,7]; patients diagnosed with AF have a 4- to 5-fold increased risk of ischemic stroke compared with patients without AF [8–10]. A main goal of anticoagulant therapy is to prevent or reduce the risk of thromboembolic events, and the American College of Cardiology and American Heart Association (ACC/AHA) guidelines for AF recommend the use of oral anticoagulants (OACs) in patients with AF based on risk stratification using the CHA_2_DS_2_-VASc (congestive heart failure, hypertension, age ≥75 years, diabetes mellitus, stroke or transient ischemic attack, vascular disease, age 65 to 74 years, sex category) score for each individual patient [11]. ACC/AHA recommend direct-acting OACs (DOACs; eg, apixaban, rivaroxaban, dabigatran, and edoxaban) over warfarin for stroke prevention in AF [11]. OAC therapy has been shown to significantly decrease the risk of stroke [12,13]; however, previous research has shown that guideline-concordant anticoagulant use remains suboptimal [14]. A US-based registry study found that approximately half of patients with a moderate to high risk of stroke received guideline-based treatment [15]; furthermore, an analysis of administrative claims data found that over one-third of elderly veterans in the United States diagnosed with AF were not prescribed recommended anticoagulant therapy [14]. These studies underscore the gap in evaluating the lack of concordance between clinical practice guidelines and the treatment of AF.

Factors describing the consistently large proportions of untreated patients with AF have not been well characterized and do not adequately describe how social factors are associated with lack of treatment. Social determinants of health (SDOH) are the conditions in which an individual is born, lives, works, and ages and include characteristics such as race/ethnicity, socioeconomic status, and residential environment [16,17]. Socioeconomic status, which embodies a person’s income, education, and employment, has been explored as associated with AF; several studies have suggested that patients with low socioeconomic status are linked to higher incidences of AF, poorer prognoses, and a lack of concordance with guideline-based treatment [18–20]. However, beyond socioeconomic status, little evidence is available regarding the associations between SDOH and the management of AF.

The objective of this study was to describe patient clinical factors and social determinants of health among patients recently diagnosed with AF and evaluate concordance with the guidelines in terms of OAC prescribing for stroke prevention.

## Methods

### Data source

Data were derived from EHRs collected from Advocate Aurora Health (AAH) facilities. AAH is a not-for-profit integrated healthcare organization that encompasses 26 hospitals, over 500 outpatient locations, and a clinical laboratory system in the Midwestern United States, spanning Illinois and Wisconsin. AAH serves more than 3 million distinct patients annually and covers a diverse patient population in terms of geographic location, race/ethnicity, and medical conditions. For purposes of this analysis, data were extracted from EHRs on patients with encounters in Wisconsin AAH facilities only.

This study was conducted in accordance with legal and regulatory requirements; it was reviewed by the institutional review board (IRB) of AAH prior to collection of any patient health information and was determined to be exempt by the IRB. The analysis involved anonymized structured data and contained no patient personal information; therefore, the IRB determined that informed consent from patients was not required.

### Study design and data collection

This retrospective cohort study included patients in the AAH healthcare system with a new AF diagnosis (index visit) between May 2016 and May 2020. Data on eligible patients (follow-up data) were collected for 1 year after the index visit, up to May 2021. Data were retrospectively extracted from patients with an encounter in one of AAH’s facilities in Wisconsin. Patients were included in this analysis if they were aged ≥18 years on the index date, had an index visit with a first diagnosis of AF at an AAH facility, had ≥1 AAH encounter (outpatient ambulatory visit, emergency department visit, or hospitalization) within 1 year from the index visit, and had an AF diagnosis based on predefined International Classification of Diseases, Tenth Revision (ICD-10) codes. ICD-10 codes included I48.0 (paroxysmal AF), I48.1 (persistent AF), I48.2 (chronic AF), I48.3 (typical atrial flutter), I48.4 (atypical atrial flutter), and I48.91 (unspecified AF).

Patients were excluded if they had a history of rheumatic mitral valvular heart disease or cardiac valve replacement/transplant, or hip/knee replacement, in the 6 months prior to the index date. Patients were also excluded if they became pregnant, had a venous thromboembolism, hip/knee replacement, or died during the study period.

Detailed patient-level information on demographics, clinical characteristics, comorbidities, medications, and SDOH and associated behaviors were extracted from EHRs. The primary endpoint was the proportion of patients who were prescribed an OAC within 1 year of AF diagnosis. For those who did receive an OAC prescription, the class of OAC (DOAC or vitamin K antagonist [warfarin]) and time to prescription within 1 year of the index visit were noted. The primary endpoint (OAC prescription) was evaluated at 1 year. However, as secondary outcomes OAC prescriptions were evaluated at three additional timepoints: (1) the time of diagnosis, (2) 14 days from diagnosis, and (3) at 90 days from diagnosis. When examining associations with SDOH domains, this was completed for the primary end point (OAC prescription at 1 year) and for the three secondary endpoints (OAC prescription at diagnosis, OAC prescription at 14 days from diagnosis, and OAC prescription at 90 days from diagnosis). All SDOH in EHRs were patient-reported except alcoholism, which is based on *ICD-10* codes. Area deprivation index (ADI), updated in 2018, is reported as a composite score ranging from 1 (least socioeconomically disadvantaged) to 100 (most socioeconomically disadvantaged) and comprises 17 education, employment, housing quality, and poverty measures originally drawn from long-form US Census data and periodically updated [21–22]. Patient data were not linkable to ADI for Illinois; therefore, only patients with encounters in Wisconsin were used for this analysis.

### Data analysis

Descriptive statistics were calculated for all variables and presented overall and by group (prescribed vs nonprescribed) using means and standard deviations (SDs) for continuous variables and counts and percentages for categorical or ordinal variables. Comparisons between groups were made using χ^2^ tests for categorical data and Student *t* tests or Mann-Whitney tests as appropriate for parametric and nonparametric distributions, respectively, for all continuous data. Multivariable regression models were used to explore the associations between SDOH and the outcomes of interest. Covariates of interest were added to models to adjust for possible confounding by demographic, clinical, and SDOH characteristics; these included insurance status, marital status, preferred language, race/ethnicity, religion, history of alcoholism, smoking status, age, sex, hypertension, stroke, transient ischemic attack, myocardial infarction, chronic kidney disease, congestive heart failure, venous thromboembolism, diabetes, diagnosing provider, CHA_2_DS_2_-VASc score (for scores ≥2, an OAC prescription is recommended), modified HAS-BLED score (hypertension, abnormal liver/renal function, stroke history, bleeding history or predisposition, elderly, drug/alcohol use [international normalized ratios were not captured]; for scores ≥3, caution is warranted when prescribing an OAC), [23] and ADI by clusters. Given the low proportions of missing data from the EHRs used in this study, no imputation of missing data was performed. Any cases of missing values for variables included in the model (eg, race/ethnicity) were excluded from the analytic sample for that analysis. Analyses were completed using SAS software 9.4 (SAS Institute Inc, Cary, NC).

## Results

### Patient baseline characteristics

**Fig 1:**
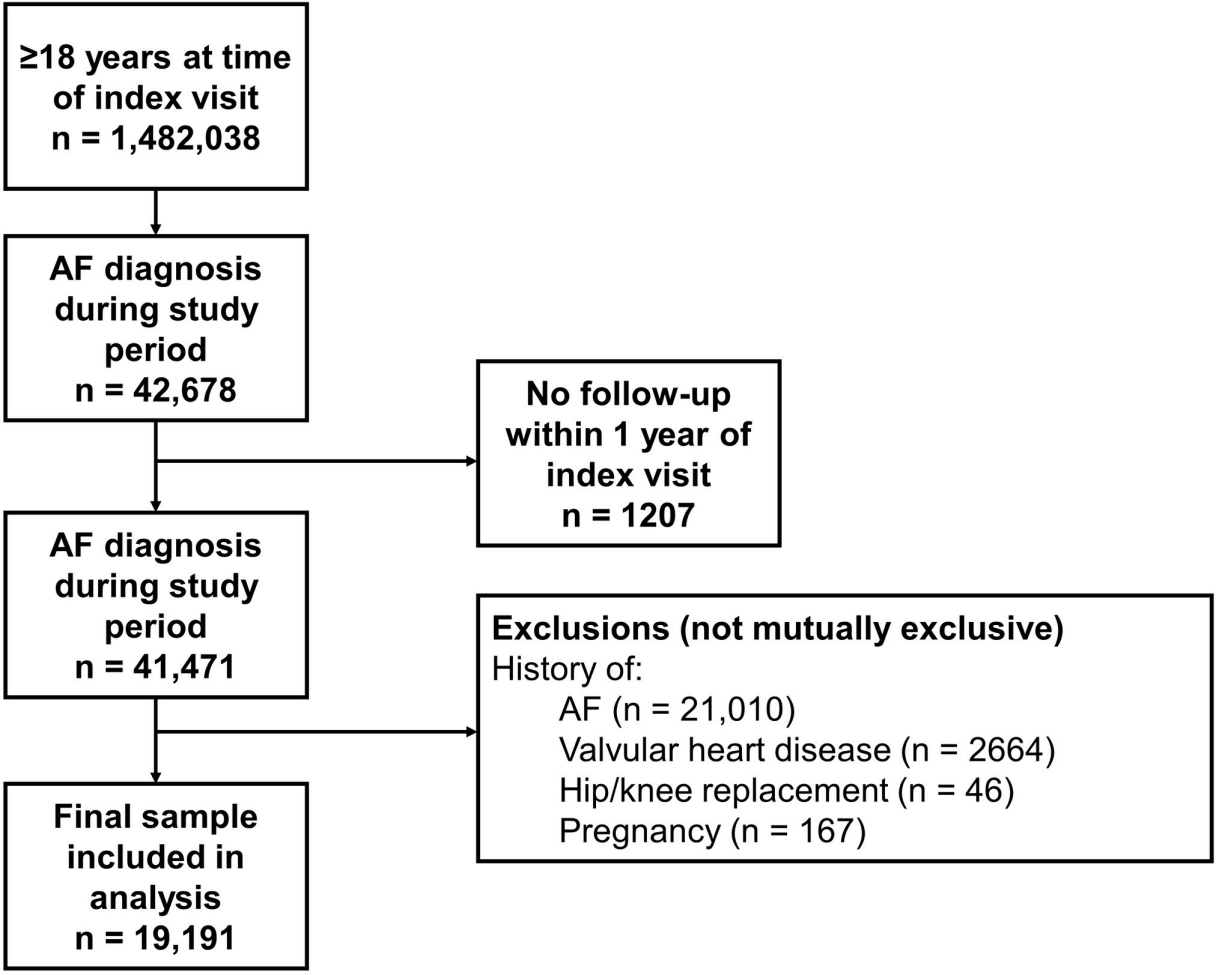
Patient flow diagram. AF, atrial fibrillation; VTE, venous thromboembolism.

A total of 16,656 patients with newly diagnosed AF between May 2016 and May 2020 were included in the study (**Fig 1**). The mean (SD) age of all patients at the time of diagnosis was 70.4 (12.8) years and the majority were male (56.4%), White (91.3%), and prescribed aspirin (67.8%) (**Table 1**). Hypertension was the most common comorbidity (59.3%), followed by coronary artery disease (25.0%) and diabetes (21.9%). The mean (SD) CHA_2_DS_2_-VASc score was 2.7 (1.6), and 75.3% of patients had a score ≥2. Patients’ mean (SD) HAS-BLED score was 1.4 (0.9), and 11.0% had a score ≥3.

**Table 1:** Baseline characteristics of patients diagnosed with atrial fibrillation between 2016 and 2020.

Characteristic	Not prescribed anticoagulant within 1 year N = 5,758 (34.6%)	Prescribed anticoagulant within 1 year N = 10,898 (65.4%)	All patients N = 16,656	*P*-Value
**Age in years, mean (SD)**	69.2 (14.7)	71.1 (11.7)	70.4 (12.8)	<0.01
<75 years old, n (%)	3,609 (62.7%)	6,579 (60.4%)	10,188 (61.2%)	<0.01
≥75 years old, n (%)	2,149 (37.3%)	4,319 (39.6%)	6,468 (38.8%)	
**Female, n(%)**	2,537 (44.1%)	4,719 (43.3%)	7,256 (43.6%)	0.35
**Race/Ethnicity, n(%)**				
White	5,202 (90.3%)	10,004 (91.8%)	15,206 (91.3%)	0.01
Black	291 (5.1%)	498 (4.6%)	789 (4.7%)	
Hispanic/Latino	165 (2.9%)	258 (2.4%)	423 (2.5%)	
Asian	100 (1.7%)	138 (1.3%)	238 (1.4%)	
**Non-English speaking, n(%)**	142 (2.5%)	244 (2.2%)	386 (2.3%)	0.35
**Comorbidities at diagnosis and 6 months prior, n (%)**				
Hypertension	3,092 (53.7%)	6,776 (62.2%)	9,868 (59.3%)	<0.01
Stroke	286 (5.0%)	645 (5.9%)	931 (5.6%)	0.01
TIA	85 (1.5%)	205 (1.9%)	290 (1.7%)	0.06
Coronary artery disease	1,481 (25.7%)	2,674 (24.5%)	4,155 (25.0%)	0.09
Chronic kidney disease	376 (6.5%)	727 (6.7%)	1,103 (6.6%)	0.73
Myocardial infarction	279 (4.9%)	439 (4.0%)	718 (4.3%)	0.01
Heart Failure	691 (12.0%)	1,875 (17.2%)	2,566 (15.4%)	<0.01
Venous thromboembolism	33 (0.6%)	92 (0.8%)	125 (0.8%)	0.05
Diabetes	1,056 (18.3%)	2,596 (23.8%)	3,652 (21.9%)	<0.01
**Aspirin use, n (%)**	3,941 (68.4%)	7,348 (67.4%)	11,289 (67.8%)	0.18
**Diagnosis setting, n (%)**				
Outpatient	5,110 (96.9%)	9,364 (96.8%)	14,474 (96.8%)	0.85
Inpatient^a^	165 (3.3%)	308 (3.2%)	473 (3.2%)	
**Provider specialty at diagnosis, n (%)**				
Primary care^b^	3,533 (61.4%)	6,585 (60.4%)	10,118 (60.8%)	<0.01
Cardiology	779 (13.5%)	1,220 (11.2%)	1,999 (12.0%)	
Emergency Medicine	4 (0.1%)	24 (0.2%)	28 (0.2%)	
Neurology	63 (1.1%)	57 (0.5%)	120 (0.7%)	
Other^c^	1,379 (24.0%)	3,012 (27.6%)	4,391 (26.4%)	
**CHA2DS2-VASc score at DX, mean (SD)**	2.5 (1.6)	2.8 (1.5)	2.7 (1.6)	<0.01
<2**, n (%)**	1,833 (31.8%)	2,290 (21.0%)	4,123 (24.8%)	<0.02
≥2**, n (%)**	3,925 (68.2%)	8,608 (79.0%)	12,533 (75.3%)	
**HAS-BLED at DX score, mean (SD)**	1.3 (0.9)	1.4 (0.9)	1.4 (0.9)	<0.01
0, n (%)	1,177 (20.4%)	1,868 (17.1%)	3,045 (18.3%)	<0.01
1–2, n (%)	3,986 (69.2%)	7,788 (71.5%)	11,774 (70.7%)	
≥3, n (%)	595 (10.3%)	1,242 (11.4%)	1,837 (11.0%)	
**Diagnosis Year, n (%)**				
2016	982 (17.1%)	1,526 (14.0%)	2,508 (15.1%)	<0.01
2017	1,398 (24.3%)	2,515 (23.1%)	3,913 (23.5%)	
2018	1,367 (23.7%)	2,914 (26.7%)	4,281 (25.7%)	
2019	1,515 (26.3%)	2,926 (26.9%)	4,441 (26.7%)	
2020^d^	496 (8.6%)	1,017 (9.3%)	1,513 (9.1%)	

CHA_2_DS_2_-VASc, congestive heart failure, hypertension, age ≥75 years, diabetes mellitus, stroke or transient ischemic attack, vascular disease, age 65 to 74 years, sex category; HAS-BLED, hypertension, abnormal liver/renal function, stroke history, bleeding history or predisposition, elderly, drug/alcohol use.

^a^ Includes admission, emergency department, and observation.

^b^ Includes internal medicine, geriatrics, family medicine, and pediatrics.

^c^ Includes nurse practitioner/physician assistant (unspecified), other, or missing.

^d^ Includes data only through May 2020.

### OAC prescribing

A total of 10,898 (65.4%) patients were prescribed an OAC within the first year of diagnosis (**Table 1**). Of these patients, 24.2% were prescribed the OAC at the time of diagnosis and 37.7% were prescribed it within 14 days; and 55.2% were prescribed it within 90 days of diagnosis (**S1 Appendix**).

**Fig 2:**
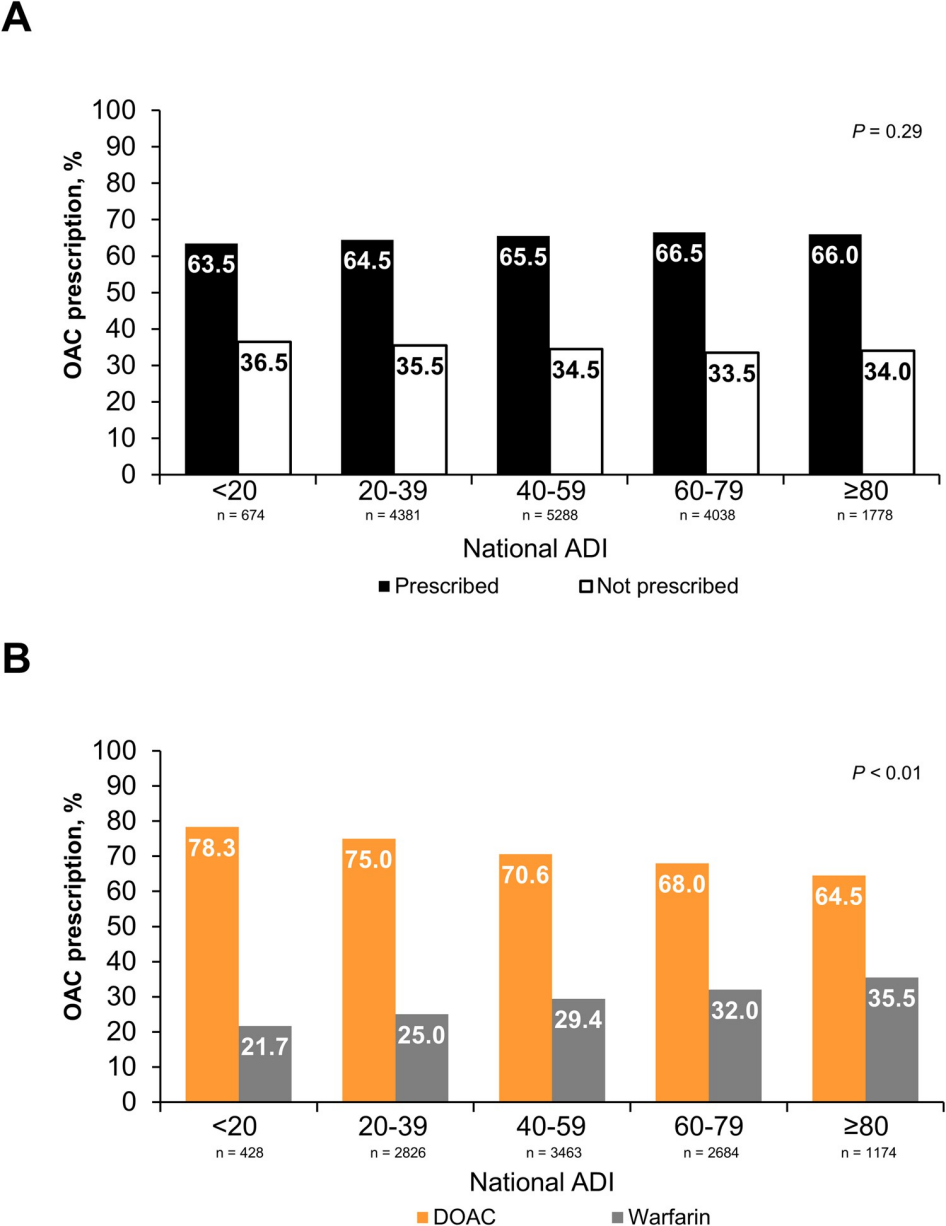
Class of anticoagulant prescribed and aspirin use by CHA_2_DS_2_-VASc score^a^. CHA_2_DS_2_-VASc, congestive heart failure, hypertension, age ≥75 years, diabetes mellitus, stroke or transient ischemic attack, vascular disease, age 65 to 74 years, sex category; DOAC, direct-acting oral anticoagulant; OAC, oral anticoagulant. All study patients with scores between 2–8 were included in this analysis (N = 12,533). ^a^ Anticoagulant care is recommended for CHA_2_DS_2_-VASc scores ≥2.

Among patients with a CHA_2_DS_2_-VASc scores ≥2, 8,608 (79.0%) received a prescription (**Table 1**). **Fig 2** further describes OAC prescribing among the subgroup of patients with CHA_2_DS_2_-VASc scores ≥2 (N = 12,533). Overall, the proportion of patients who did not receive an OAC decreased very slightly from 31.0% among patients with a score of 2 to 27.3% among patients with a score of 8. As CHA_2_DS_2_-VASc scores increased, the proportion of patients receiving DOAC prescriptions decreased, whereas the proportion of warfarin and aspirin prescriptions increased.

**Fig 3:**
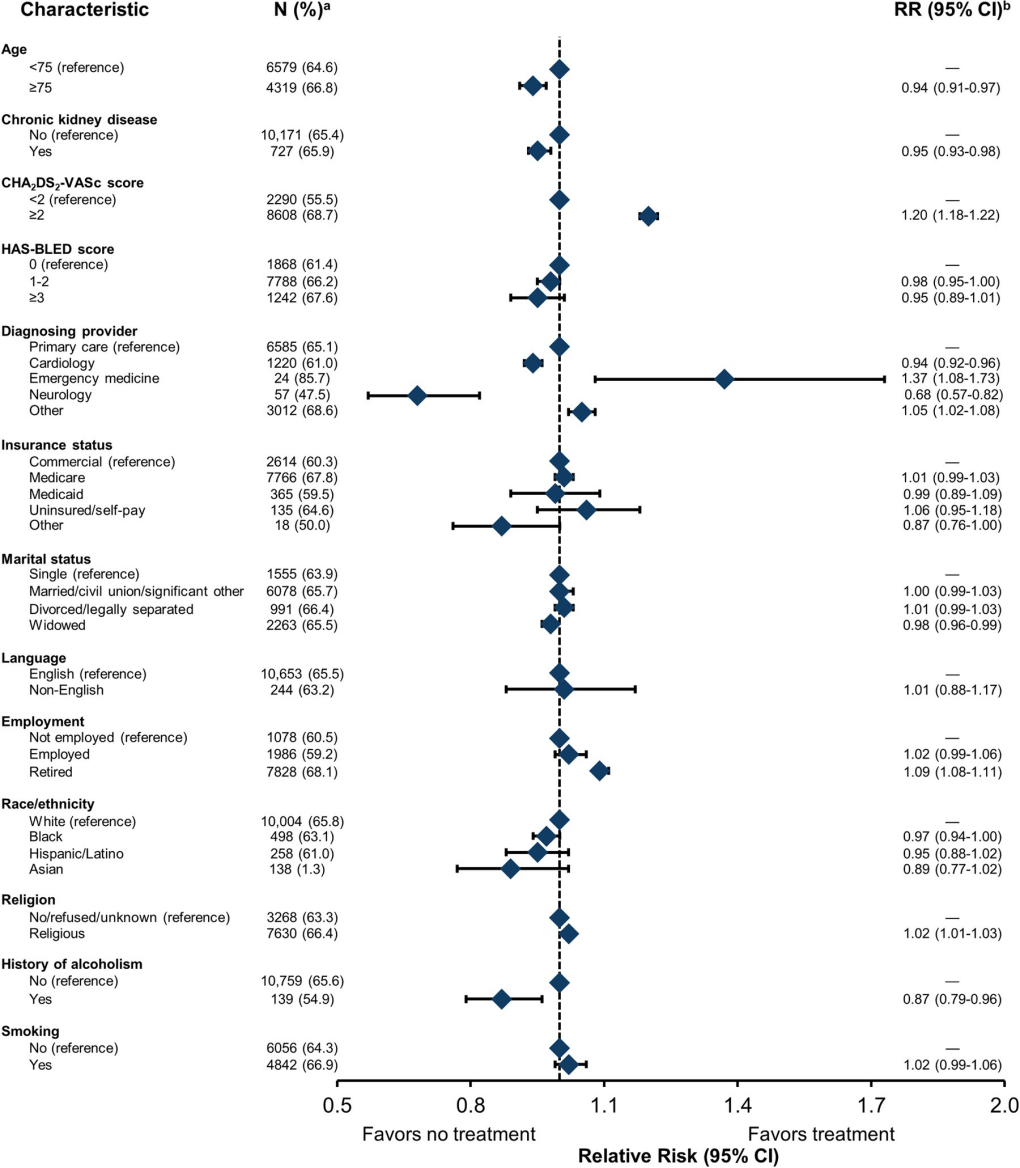
Relationship between diagnosing and prescribing providers among patients recently diagnosed with AF. AF, atrial fibrillation; OAC, oral anticoagulant. All study patients were included in this analysis (N = 16,656). ^a^ Primary care includes internal medicine, geriatrics, family medicine, and pediatrics. ^b^ Other includes nurse practitioner/physician assistant (unspecified), other, or missing.

Primary care providers most frequently diagnosed patients with AF (60.8%), followed by providers in cardiology (12.0%) (**Table 1**). **Fig 3A** visualizes the proportion of patients by diagnosing provider and by prescribing provider. **Fig 3B** shows that of 10,118 patients diagnosed with AF in primary care, 42.6% were prescribed an OAC by primary care providers, 34.9% were not prescribed an OAC, and the remaining patients were prescribed an OAC by other specialties. Of 1999 patients diagnosed with AF in cardiology 12.0% were prescribed an OAC by cardiologists, 39% were not prescribed an OAC, and the remaining patients were prescribed an OAC by other specialties.

**Fig 4:**
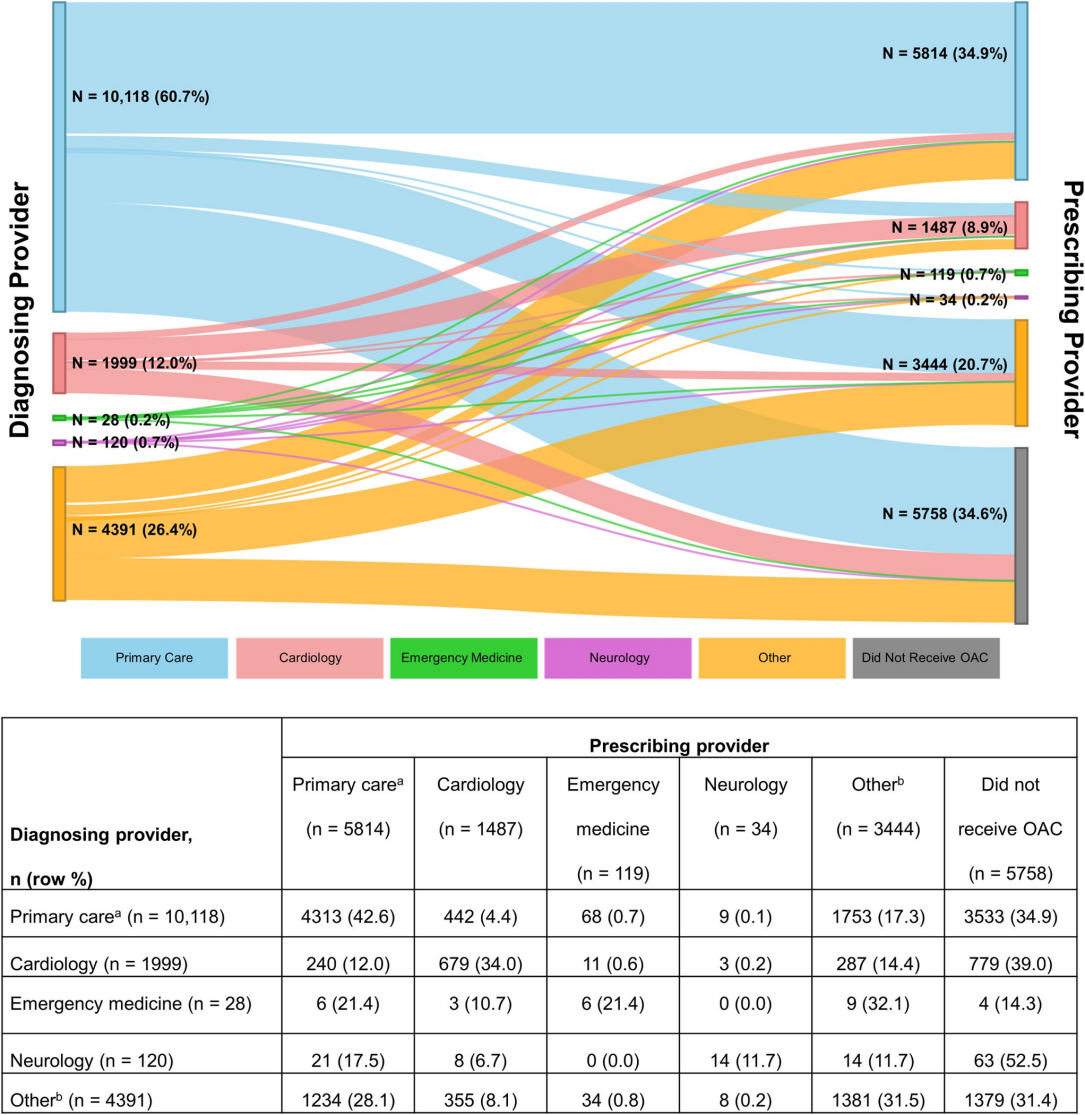
National ADI^a^ among patients diagnosed with AF and (A) anticoagulant prescription and (B) class prescribed. ADI, area deprivation index; AF, atrial fibrillation; DOAC, direct-acting oral anticoagulant; OAC, oral anticoagulant. Fig 4A includes all patients (N = 16,159) included in this study who had an ADI score. Fig 4B was limited to patients who were prescribed an OAC and had an ADI score (N = 10,575 patients). ^a^ ADI scores range from 1 (least socioeconomically disadvantaged) to 100 (most socioeconomically disadvantaged) and is missing for some patients.

The proportion of patients who were prescribed an OAC was similar (63.5% among the least disadvantaged population vs 66.0% among the most disadvantaged; *P* = 0.29) across national ADI quintiles (**Fig 4A**). As the national ADI score increased, the proportion of patients receiving a DOAC vs warfarin decreased from 78.3% for ADI <20 (least disadvantaged) to 64.5% for ADI ≥80 (most disadvantaged; *P*<0.01; **Fig 4B**).

### Social and clinical factors associated with OAC prescribing

**Fig 5:**
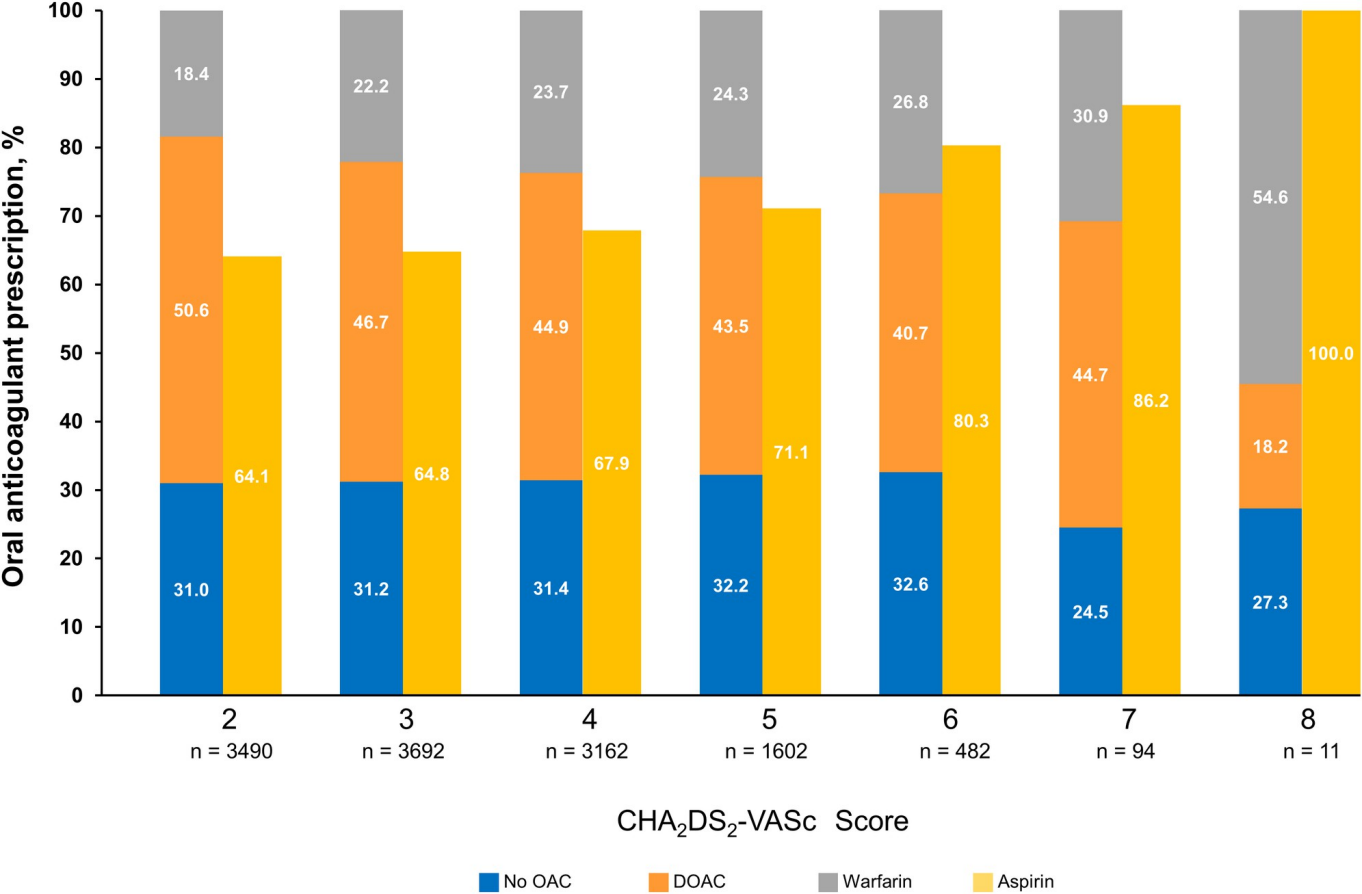
Association between SDOH domains, associated behaviors, and anticoagulant prescription within 1 year of AF diagnosis. AF, atrial fibrillation; CHA_2_DS_2_-VASc, congestive heart failure, hypertension, age ≥75 years, diabetes mellitus, stroke or transient ischemic attack, vascular disease, age 65 to 74 years, sex category; HAS-BLED, hypertension, abnormal liver/renal function, stroke history, bleeding history or predisposition, elderly, drug/alcohol use; RR, relative risk; SDOH, social determinants of health. A total of 16,124 patients had data on all independent variables and were included in this regression model. ^a^ Percentage reflects the proportion of the population within a category. ^b^ Covariates in the model included insurance status, marital status, preferred language, race/ethnicity, religion, history of alcoholism, smoking status, age, sex, hypertension, stroke, transient ischemic attack, myocardial infarction, chronic kidney disease, congestive heart failure, venous thromboembolism, diabetes, diagnosing provider, CHA_2_DS_2_-VASc score, HAS-BLED score, and cluster for area deprivation index.

In fully adjusted models, clinical factors associated with not receiving an OAC prescription included being ≥75 years (0.94 [0.91–0.97] vs <75 years), having chronic kidney disease (0.95 [0.93–0.98] vs no disease), or being diagnosed by cardiology (0.94 [0.92, 0.96]) vs primary care) or neurology (0.68 [0.57, 0.82] vs primary care), while being diagnosed by emergency medicine was associated with an increased likelihood of receiving an OAC prescription (1.37 [1.08, 1.73]) vs primary care; **Fig 5**). In terms of social factors, patients were less likely to be prescribed an OAC if they were widowed (0.98 [0.96–0.99] vs single) or had a history of alcoholism (0.87 [0.79–0.96] vs no history) (**Fig 5**).

### Social and clinical factors associated with OAC class

**Fig 6:**
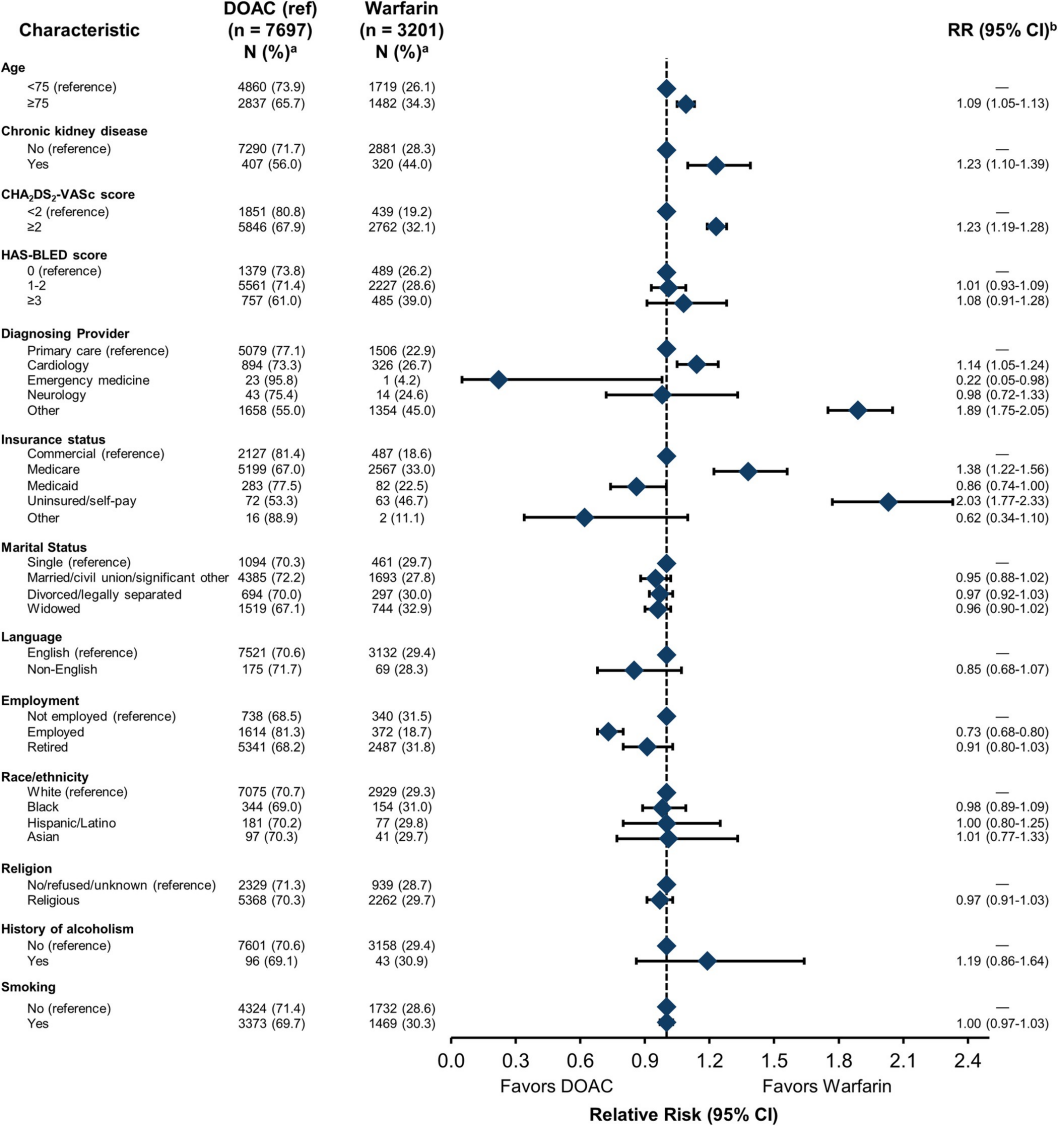
Association between SDOH domains, associated behaviors, and class of anticoagulant prescribed (DOAC vs warfarin). CHA_2_DS_2_-VASc, congestive heart failure, hypertension, age ≥75 years, diabetes mellitus, stroke or transient ischemic attack, vascular disease, age 65 to 74 years, sex category; DOAC, direct-acting oral anticoagulant; HAS-BLED, hypertension, abnormal liver/renal function, stroke history, bleeding history or predisposition, elderly, drug/alcohol use; RR, relative risk; SDOH, social determinants of health. This analysis was limited to patients who were prescribed an OAC (N = 10,575), of which 10,558 patients had data on all independent variables and were included in these regression models. ^a^ Percentage reflects the proportion of the population within a category. ^b^ Covariates in the model included insurance status, marital status, preferred language, race/ethnicity, religion, history of alcoholism, smoking status, age, sex, hypertension, stroke, transient ischemic attack, myocardial infarction, chronic kidney disease, congestive heart failure, venous thromboembolism, diabetes, diagnosing provider, CHA_2_DS_2_-VASc score, HAS-BLED score, and cluster for area deprivation index.

Of 10,898 patients who received an OAC and were included in fully adjusted models, 7697 (70.6%) received a DOAC and 3201 (29.4%) received warfarin (**Fig 6**). In fully adjusted models, clinical factors associated with receiving a prescription for warfarin (compared to a DOAC) included being ≥75 years (1.09 [1.05–1.13] vs aged <75 years), having chronic kidney disease (1.23 [1.10–1.39] vs no disease), and being diagnosed within cardiology or other specialties (1.14 [1.05–1.24] and 1.89 [1.75–2.05], respectively, compared to primary care.

Among social factors, having Medicare or self-pay insurance (1.38 [1.22–1.56] and 2.03 [1.77–2.33], respectively, vs commercial insurance) was associated with a Warfarin prescription. While patients who were employed (0.73 [0.68–0.80] vs unemployed) and had Medicaid (0.86 [0.74–1.00] vs commercial insurance) were less likely to receive warfarin.

## Discussion

Using EHR data from a large healthcare system, we found a gap in OAC prescribing among patients newly diagnosed with AF. Even after a full year of diagnosis, only two thirds of patients received a prescription as recommended in clinical practice guidelines. Several factors were associated with not receiving an OAC prescription. Clinical factors included older age, a documented diagnosis of chronic kidney disease, and being diagnosed within cardiology or neurology compared to primary care. While social and behavioral factors included history of alcoholism and being widowed. As expected, there was a strong association between OAC prescribing and greater CHA_2_DS_2_-VASc scores, even though the gap in prescribing persisted among patients with high scores.

This study narrows the current literature gap in anticoagulant initiation in patients with documented AF. Patients exhibiting higher-risk clinical factors such as chronic kidney disease or increased age were less likely to receive OAC prescriptions, despite evidence demonstrating the beneficial use of OACs [23–26]. Although the decision to prescribe an OAC for patients with AF was strongly associated with CHA_2_DS_2_-VASc score, our analysis reaffirms that overall OAC initiation remains suboptimal in patients diagnosed with AF; one-third of patients did not receive a prescription within the first year of diagnosis, including >20% of high-risk patients (CHA_2_DS_2_-VASc score ≥2). These results are consistent with data from the United States and other, international studies suggesting that stroke prevention in patients with AF remains discordant with evidence-based guidelines. Despite guidelines recommending initiation of anticoagulant therapy in high-risk patients with AF [11], approximately 33% to 50% of patients in the United States do not receive anticoagulant treatment after their diagnosis [14,15]. Studies from Denmark [27], Western Australia [28], and 2 global registries [29,30] also demonstrated the underuse of anticoagulants in patients with AF, suggesting that this trend extends outside the United States. Our results add to the current evidence-based literature regarding anticoagulants being underused among patients with AF and highlight the need for strategies to increase anticoagulant initiation, particularly in primary care settings, where most of these patients seem to be diagnosed and managed.

An interesting observation from our study was the large proportion of patients utilizing aspirin concurrently with either a DOAC or warfarin. These findings are not consistent with current evidence-based guidelines, which removed aspirin as a first-line therapy for atrial fibrillation in 2014 [31]. Further recent evidence has suggested that concurrent aspirin use may be more harmful in patients diagnosed with AF, regardless of their anticoagulant treatment. A retrospective cohort study comparing concurrent aspirin and DOAC therapy vs DOAC alone found that concomitant use was associated with increased risk of major adverse cardiac and bleeding events [32]. These patients had similar mean (SD) baseline CHA_2_DS_2_-VASc scores as our analysis (2.9 [1.8] vs 2.7 [1.6], respectively). Data from a post-hoc analysis of the SPORTIF III and V trials found that higher bleeding risk in patients treated with warfarin with poor anticoagulation control was greater with concomitant use of aspirin [33]. However, despite guideline recommendations and evidence suggesting otherwise, aspirin is still continued as a method for stroke prevention in AF patients [34–36]. Our results highlight the disconnect between evidence-based guidelines and what is occurring in clinical practice, suggesting a potential lack of awareness of policy changes that may be occurring with healthcare providers.

This study was among the first to explore the potential impact of a diverse range of clinical factors, social factors, and associated behaviors on OAC prescription patterns. Although most patients in our study population were White (91.3%) and the observed disparities were relatively small, there was a marginal trend toward non-White patients being less likely to be prescribed an OAC than White patients. These findings are consistent with previous studies in the United States in which Black patients were less likely to be prescribed anticoagulants 1 year after AF diagnosis [37–40]. There are several nonmedical factors, such as patient perceptions, treatment adherence, and trust in the medical community, which can play a role in racial/ethnic healthcare disparities that this study cannot address; further investigation into patient and provider perceptions is warranted. Similarly, our data indicating a linear relationship with worsening ADI and increasing warfarin prescriptions are consistent with other recent studies showing how neighborhood-based health inequities affects receipt of OAC prescriptions [41]. To our knowledge, this analysis is novel in its approach of examining the association between patient behaviors, such as alcoholism, and OAC prescribing patterns after the diagnosis of AF. Patients with a history of alcoholism were overall less likely to be prescribed an OAC. History of alcoholism may increase bleeding risk, and providers may therefore hesitate to prescribe an OAC [42,43]. However, alcoholism is an SDOH-associated behavior and may be a proxy for unmeasured SDOHs [44]. Substance use disorders are often stigmatized conditions in medicine, and the attitude of healthcare professionals toward patients with these conditions is often negative [45]. While numerous studies have explored the impact that SDOH such as socioeconomic status or race/ethnicity have in the treatment of AF, our study highlights the need for additional research in other SDOH where implicit bias can play a large role in the treatment patients receive.

These findings also highlight the importance of the diagnosis setting, which can have significant clinical and public health implications. A large proportion of patients in our study were diagnosed with AF by primary care providers, who are often the first point of contact for many patients and are ideally suited for screening AF since they are within a setting of more preventive care. Although most patients with AF in our study were diagnosed in the primary care setting, over a third of these patients (34.9%) did not receive an OAC prescription within 1 year of their diagnosis. The results of our study underscore the importance of further education, training, and other interventions to increase the uptake of anticoagulants, particularly in primary care settings where many of these patients may receive an AF diagnosis.

Our observational data evaluating factors associated with OAC prescribing for patients diagnosed with AF should be interpreted in the context of a few limitations. Although the study included a large patient population, data were limited to a single healthcare system, and our results may not be generalizable to other health systems. Patients may have been diagnosed with AF at AAH but received a prescription from another institution; to improve the accuracy of our results, the patient sample was limited to those who had ≥2 encounters during the study period. However, this does not guarantee excluding any patient who may have received care elsewhere. To ensure that the definitions of SDOH were consistent, we only included variables commonly found in EHR data; similarly, our results are limited to whether a patient was prescribed an OAC, and any potential information on patient adherence was not included. Our analysis examined associations between SDOH and the prescribing patterns for OACs only; any statements suggesting a causal link between SDOH and provider decision-making are beyond the scope of this study.

## Conclusion

In conclusion, EHR data from a large healthcare system in the Midwestern United States indicated a gap in evidence-based management of AF, in which one-third of patients were not prescribed an OAC within 1 year of diagnosis. Clinical characteristics, such as older age and having chronic kidney disease, are strongly associated with not receiving a prescription despite clinical practice guideline recommendations. While a larger proportion of patients were diagnosed with AF and prescribed an OAC in primary care compared to other specialties such as cardiology, primary care providers are still potentially undertreating patients a year after diagnosis, underlining the need for further evidence-based education in this setting. The results of this study highlight opportunities to improve care within healthcare systems and suggest that further training in the guideline-concordant management of AF and the effects of SDOH is warranted among healthcare providers.

## Supporting information

S1 Appendix Association between SDOH domains and associated behaviors and time of receiving an anticoagulant prescription. (DOCX)
