# Supplementary material for: Associations between clinical and social factors and anticoagulant prescription among patients with atrial fibrillation: A retrospective cohort study from a large healthcare system
Source: PLoS One. 2023 Aug 10;18(8):e0289708. doi: 10.1371/journal.pone.0289708 (PMC10414629; doi:10.1371/journal.pone.0289708)
Supplement: S1 Appendix — (DOCX) [file pone.0289708.s001.docx]

**Appendix. Association between SDOH domains and associated behaviors and time of receiving an anticoagulant prescription.**

| **Characteristic^a^** | **OAC Prescribed at Diagnosis**  **N=3,916 (24.2%)** | **OAC Prescribed by Day 14**  **N=6,095 (37.7%)** | **OAC Prescribed by Day 90**  **N=8,921 (55.2%)** |
| --- | --- | --- | --- |
|  | **Fully adjusted RR (95%CI)** | **Fully adjusted RR (95%CI)** | **Fully adjusted RR (95%CI)** |
| **Age** |  |  |  |
| <75 years old | Reference | Reference | Reference |
| ≥75 years old | 0.93 (0.85-1.01) | 0.91 (0.85-0.97) | 0.91 (0.88-0.95) |
| **Chronic Kidney Disease** |  |  |  |
| No | Reference | Reference | Reference |
| Yes | 0.96 (0.84-1.11) | 0.95 (0.91-0.99) | 0.90 (0.88-0.93) |
| **CHA2DS2-VASc score** |  |  |  |
| ≤2 | Reference | Reference | Reference |
| >2 | 1.28 (1.12-1.46) | 1.23 (1.13-1.33) | 1.18 (1.16-1.20) |
| **HAS-Bled Score** |  |  |  |
| 0 | Reference | Reference | Reference |
| .1-2 | 0.74 (0.68-0.81) | 0.86 (0.83-0.90) | 0.99 (0.97-1.02) |
| ≥3 | 0.56 (0.52-0.61) | 0.74 (0.68-0.80) | 0.98 (0.94-1.03) |
| **Diagnosing Provider** |  |  |  |
| Primary care | Reference | Reference | Reference |
| Cardiology | 1.01 (0.92-1.10) | 0.91 (0.87-0.96) | 0.93 (0.90-0.95) |
| Emergency Medicine | 1.56 (1.10-2.21) | 2.20 (1.77-2.75) | 1.60 (1.29-1.99) |
| Neurology | 0.67 (0.43-1.06) | 0.59 (0.44-0.81) | 0.66 (0.53-0.81) |
| Other* | 0.68 (0.66-0.70) | 0.92 (0.90-0.94) | 1.02 (1.00-1.03) |
| **Insurance status** |  |  |  |
| Commercial | Reference | Reference | Reference |
| Medicare | 1.03 (0.98-1.09) | 1.02 (0.94-1.11) | 1.01 (0.98-1.05) |
| Medicaid | 1.03 (0.98-1.08) | 0.96 (0.90-1.03) | 0.99 (0.89-1.10) |
| Uninsured/ self-pay | 1.21 (1.00-1.46) | 1.17 (0.99-1.39) | 1.12 (0.93-1.35) |
| Other | 1.13 (0.69-1.84) | 1.01 (0.81-1.27) | 0.93 (0.77-1.13) |
| **Marital status** |  |  |  |
| Single | Reference | Reference | Reference |
| Married/ Civil union/ significant other | 1.01 (0.95-1.08) | 1.01 (0.94-1.08) | 1.00 (0.97-1.02) |
| Divorced/ legally separated | 1.03 (0.98-1.09) | 1.01 (0.93-1.09) | 0.98 (0.95-1.00) |
| Widowed | 0.98 (0.87-1.10) | 0.98 (0.92-1.04) | 0.96 (0.95-0.97) |
| **Language** |  |  |  |
| English | Reference | Reference | Reference |
| Non-English | 1.08 (0.69-1.69) | 1.09 (0.78-1.51) | 1.06 (0.84-1.34) |
| **Employment** |  |  |  |
| Not Employed | Reference | Reference | Reference |
| Employed | 1.07 (1.00-1.14) | 1.10 (1.05-1.14) | 1.05 (1.01-1.09) |
| Retired | 1.13 (1.02-1.26) | 1.17 (1.10-1.24) | 1.12 (1.11-1.13) |
| **Race/Ethnicity** |  |  |  |
| White | Reference | Reference | Reference |
| Black | 0.97 (0.91-1.04) | 0.88 (0.80-0.97) | 0.95 (0.93-0.96) |
| Hispanic/Latino | 0.82 (0.56-1.21) | 0.82 (0.61-1.10) | 0.91 (0.79-1.05) |
| Asian | 0.94 (0.64-1.37) | 0.85 (0.61-1.18) | 0.87 (0.72-1.04) |
| **Religion** |  |  |  |
| No/ Refused/ Unknown | Reference | Reference | Reference |
| Religious | 0.97 (0.88-1.05) | 0.99 (0.95-1.02) | 1.01 (0.99-1.04) |
| **Alcoholism** |  |  |  |
| No | Reference | Reference | Reference |
| Yes | 0.78 (0.67-0.92) | 0.79 (0.66-0.95) | 0.80 (0.73-0.89) |
| **Smoking** |  |  |  |
| No | Reference | Reference | Reference |
| Yes | 0.96 (0.89-1.04) | 1.00 (0.95-1.05) | 1.02 (0.98-1.06) |

ADI, area deprivation index; CHA2DS2-VASc, congestive heart failure, hypertension, age ≥75 years, diabetes mellitus, stroke or transient ischemic attack, vascular disease, age 65 to 74 years, sex category; HAS-BLED, hypertension, abnormal liver/renal function, stroke history, bleeding history or predisposition, elderly, drug/alcohol use; OAC, oral anticoagulant; RR, relative risk; SDOH, social determinants of health; TIA, transient ischemic attack; VTE, venous thromboembolism.

A total of 16,124 patients had data on all independent variables and were included in these regression models.

Adjusted for age, chronic kidney disease, cha2ds2vascat, has-bled, diagnosing provider, insurance status, marital status, language, employment, race/ethnicity, religion, alcoholism, smoking, sex, hypertension, stroke, TIA, myocardial infarction, congestive heart failure, VTE, diabetes, and cluster for ADI.

^a^ Percentage reflects the proportion of the population within a category.
